# A Short, Multimodal Activity Break Incorporated Into the Learning Context During the Covid-19 Pandemic: Effects of Physical Activity and Positive Expressive Writing on University Students' Mental Health—Results and Recommendations From a Pilot Study

**DOI:** 10.3389/fpsyg.2021.645492

**Published:** 2021-08-12

**Authors:** Verena Marschin, Cornelia Herbert

**Affiliations:** Department of Applied Emotion and Motivation Psychology, Institute of Psychology and Education, Ulm University, Ulm, Germany

**Keywords:** Covid-19, physical inactivity, mental health, stress, university, physical activity program, positive expressive writing, cognitive intervention

## Abstract

Physical inactivity, sedentary behavior and mental ill health, due to high levels of perceived stress or self-reported depressive symptoms, are highly prevalent among university students. There are concerns that these behaviors and mental symptoms have significantly increased during the current Covid-19 pandemic, partly because academic life has changed considerably from face-to-face communication to e-learning and studying at home. Self-regulation and physical activity are hard to maintain during pandemic lockdowns. Short activity breaks could be helpful to avoid physical inactivity and sustain mental health. The breaks should comprise short and easy-implementable physical activity exercises that can be integrated into the learning context. Moreover, cognitive interventions, such as writing about positive events and feelings might help as coping strategy for self-regulation during study breaks. This study investigated and compared the effects of a physical activity intervention and a cognitive intervention (positive expressive writing) on mental health among university students. Both interventions are particularly suitable for use at home. *N* = 20 university students, studying in Germany, were assigned to a physical activity group or a cognitive intervention group. The physical activity intervention consisted of a mix of physical exercises including endurance exercises, muscular strength, relaxation, and ballroom dance movements. The interventions were carried out guided, once a week, for 5–10 mins at the beginning of classes. The effects of *group* × *time* showed no significant interaction on self-reported perceived stress, mood, quality of life (QoL) assessed online and compared at the beginning of the term before the intervention (T0) and at the end of the term after the intervention (T3). However, the physical activity group reported a similar physical activity level per day over time, while the cognitive intervention group showed a decrease in physical activity from T0 to T3. Low-dose, short physical activity interventions as well as cognitive interventions consisting of positive expressive writing could buffer university students' perceived stress, mood, and QoL across the term. Moreover, both interventions seem to be promising in buffering the negative side effects of stress during the Covid-19 pandemic.

## Introduction

Maintenance of physical and mental health and well-being is associated with fundamental human needs across all age-groups. While bodily needs like nourishment or feelings of safety are innate, being physically active is part of human evolutionary heritage. Already during the first few hours of their life, newborns show signs of physical activity, e.g., crawling (Widström et al., 2011). Acting and “grasping the world through action, movement and physical activity” is part of a child's exploratory behavior and a necessary developmental means for brain and mental development as well. In fact, staying physically active and embodied is essential for healthy aging in general (Engeroff et al., 2018). Crucially, physical activity and feelings of safety are momentarily at risk due to the Coronavirus pandemic (Covid-19; Maugeri et al., 2020; Woods et al., 2020). Especially social distancing, caused by the pandemic societal lockdowns, is sought to lead to increased physical inactivity and mental ill health (Hall et al., 2020). Importantly, not only older age groups are affected, but the pandemic has an impact on physical activity across age groups, including those age groups, that might still be equipped with good mental and physical health (Hall et al., 2020). Therefore, it is mandatory to find psychological interventions that help people to continue to exercise, despite the demand of staying at home (Lippi et al., 2020; Woods et al., 2020).

### Physical Health and Physical (In-)Activity During Covid-19

Physical activity can be understood as an umbrella term for any kind of physical movement through the contraction and relaxation of muscles that requires energy expenditure (Caspersen et al., 1985). Physical fitness is one important aspect and dimension of physical activity. It is related to health and the ability to carry out certain physical activities. Physical fitness is often divided into health-related fitness categories like cardiovascular fitness or flexibility and skill-related fitness like balance or coordination, respectively (Caspersen et al., 1985; Corbin et al., 2000). Generally, the term “exercise” should be distinguished from physical activity as a structured and planned form of physical activity (Caspersen et al., 1985). However, the terms are not consequently used or separated in the literature and instead are often used interchangeably. The positive impact of physical activity, and of physical activity or exercise interventions in particular, on health (mental and physical) is widely supported in the scientific literature. Relationships were found, e.g., between physical activity (physical fitness in particular) and rate of morbidity for certain diseases, like coronary heart disease, cardiovascular disease or cancer, but also for all-cause mortality (Blair et al., 2001; for an overview of possible underlying mechanisms, see Mikkelsen et al., 2017). Furthermore, life-style related diseases caused by overweight, obesity or high blood pressure are negatively related to global physical activity and physical fitness among different age groups (Hansen et al., 2013). In the midst of Covid-19, the risk for these diseases is likely to increase because of physical inactivity (Lippi et al., 2020). At the same time, physical activity might serve as a buffer against negative effects of Covid-19 on the central nervous system and associated mental diseases like depression, e.g., by anti-inflammatory processes and strengthening of the immune system (Woods et al., 2020).

### Mental Health Benefits Through Physical Activity and Exercise

Not only physical but also mental health can be improved by physical activity. Generally, physical activity can predict a 2-year decrease in perceived stress (Rueggeberg et al., 2012). Psychological stress is widely distributed in societies. In a survey in Germany from the year 2016, 61% of participants reported to feel stressed, with prevalence rates increasing (Techniker Krankenkasse, 2016). Perceived stress was found to be associated with negative mental health outcomes, e.g., supporting the development of a major depression (Hammen, 2005). On top of that, stress is known to also increase the risk for developing psychophysiological/psychosomatic diseases concerning the cardiovascular, nervous, endocrine and the immune system (Cool and Zappetti, 2019). Therefore, fostering physical activity, especially among those societal groups most stressed (Rueggeberg et al., 2012), should be an important aim of health, not only in general, but particularly now during the current pandemic. Since Covid-19 is especially dangerous for people with a poor immune system (Woods et al., 2020), stress should definitely be reduced. A study by Wunsch et al. (2019) showed not only chronic exercise to be beneficial when being exposed to psychosocial stress. Positive effects were also observed after an acute bout of exercise. Mood also tended to improve after 10–30 mins of acute, but also chronic exercise, as shown in a review by Chan et al. (2019). A positive association was also found for physical activity and quality of life (QoL; Gill et al., 2013). The examination of possible moderating variables, e.g., age, intensity, length, duration and dose of exercise or physical activity, for the aforementioned association between physical activity and health variables, yielded contradictory results (e.g., Bassett-Gunter et al., 2017, versus Campbell and Hausenblas, 2009; Chan et al., 2019). Nevertheless, positive outcomes on mental health were found for varying types of sports. Chekroud et al. (2018) found a reduction in mental health burden in a cross-sectional longitudinal study with more than one million participants in the US for different types of sports, among them aerobic and anaerobic exercise, but also mindfulness exercise, that builds on the relation between body and mind (e.g., yoga or tai chi), compared to participants practicing no regular exercise or receiving no physical activity or exercise intervention. More specifically, improvements in health variables like mood, anxiety or depression could be achieved likewise through aerobic and anaerobic exercise in clinical as well as in subclinical populations (Martinsen et al., 1989a,b; for an overview, see Mikkelsen et al., 2017). Whether an additive effect of aerobic and anaerobic exercise types can be obtained, e.g., through the combination of endurance, strength, relaxation and coordination training, especially in subclinical populations, remains yet to be determined.

### Recommendations on Physical Activity to Avoid Physical Inactivity During Covid-19

Concerning reports and nation-wide surveys before the Covid-19 pandemic, only 30% of the population, as stated in a study about physical activity in Germany (Techniker Krankenkasse, 2016), reported to meet the physical activity recommendations by the World Health Organization (World Health Organization, 2010). In Spain, a large survey with more than 2000 participants found the rate to meeting these guidelines to have lowered by as much as 12% due to Covid-19 confinement (López-Bueno et al., 2020). Generally, levels of physical inactivity seem to be on the rise (World Health Organization, 2010). Following WHO‘s guidelines, adults aged 18–64 years should do at least 150 mins of moderate-intensity physical activity (e.g., housework or dancing), or alternatively 75 mins of vigorous-intensity physical activity (e.g., running or fast cycling) per week (World Health Organization, 2010). As the fourth leading case for mortality (Kohl et al., 2012), physical inactivity is a concern that has to be addressed. Distinctive to physical inactivity is a sedentary lifestyle (Tremblay et al., 2010). More than 20% of people in Germany reported to sit more than 9 h per day (Techniker Krankenkasse, 2016). Generally, people in high-income countries sit between 8 and 10 h per day on average, according to ambulatory assessment based on accelerometer-estimates (Dempsey et al., 2014). A highly sedentary lifestyle can have deleterious health effects on physical health, specifically on a person's metabolism (Tremblay et al., 2010). Moreover, sedentary behavior and anxiety, depression and well-being are correlated (Gibson et al., 2017). There is evidence, that negative effects of sedentarism on mental health can be buffered by an appropriate amount of vigorous-intensity physical activity (Gerber et al., 2014). Since this high intensity cannot be reached or attained by all people, especially with stay-at-home orders during Covid-19, this strikes the question, whether a health-buffer effect can be maintained by regular exercises whose intensity and duration per session are below the typical range of moderate to high intensity workout, i.e. so called “physical activity breaks”. These low intensity exercises might include aerobic and anaerobic exercises but their frequency per session does never exceed the daily activity level and heart rate stays at a level below 70% of the VO_2_max (Centers for Disease Control, 2008, https://www.cdc.gov). Considering the duration of physical activity, there is consensus in the literature, that also short bouts of moderate-intensity exercise (<10 mins) can already be beneficial, especially for mental health, contributing to temporary mood improvements (for an overview see Powell et al., 2019). In times of the Covid-19 pandemic, short activity breaks of low- to moderate intensity, comprising different types of exercise, that do not need much financial effort, space and time, are especially helpful (Woods et al., 2020), especially also for people without any exercise experience.

### Physical (In-)Activity and Stress Among University Students

The Covid-19 pandemic led to the isolation of many university students worldwide, with the universities having closed down completely and most lectures and seminars only presented online. Already a couple of years ago, a review by Irwin (2004) stated, that only half of university students do meet the physical activity guidelines by the American College of Sports Medicine (American College of Sports Medicine, 2000). The guidelines of the ACSM are comparable to those of the World Health Organization (2010). These guidelines are supported by only 26.7% of German university students having reported to carry out physical activity for more than 2.5 h per week, which is less compared to age associated norms outside the university context (Grützmacher et al., 2017). It can be expected that this number is even higher now because of Covid-19. On top of that, the same survey found 25.3% of students to report high levels of perceived stress and fatigue. Among these students, about one out of six showed symptoms of depression and anxiety. Stressors in this target group are particularly time and performance pressure (Grobe et al., 2018), a high workload and anxiety about the future (Jadoon et al., 2010). This has recently been supported in an RCT study among German university students (Herbert et al., 2020b). In this RCT, 19.61% of the students were found to report mild to severe depressive symptoms and they reported to sit for about 7.45 h per day on average (Herbert et al., 2020b).

In order to intervene against the aforementioned negative adverse side effects of the pandemic on physical and mental health, appropriate interventions are mandatory, especially among university students. Findings from several studies support the notion that different kinds of interventions, comprising physical components, could be beneficial, among them e.g., physical activity interventions (e.g., Baghurst and Kelley, 2014) or mindfulness based (exercise) interventions (e.g., for an overview, see Regehr et al., 2013; Galante et al., 2018). The physical and mental beneficial effects of endurance and strength exercises are well-known and supported scientifically (Kramer, 2020). Relaxation methods, e.g., qigong or Progressive Muscle Relaxation (PMR) by Jacobson (1938), showed to be beneficial regarding perceived stress, relaxation, self-acceptance and anxiety in students (Sancier and Holman, 2004; Chrisman et al., 2009; Dolbier and Rush, 2012; Hubbard and Blyler, 2016). Additionally, pure cognitive interventions, based on stress diaries or on expressive writing, could also be a promising approach (Baikie et al., 2012; Pennebaker, 2018). These cognitive interventions have shown robust effects in previous studies in clinical groups (Krpan et al., 2013). Expressive writing has been used as a successful intervention in health psychology among different age and health groups (Frisina et al., 2004, for an overview). Typically, according to standard writing protocols (Pennebaker and Beall, 1986), participants are asked to write about their most stressful negative life events repeatedly in several sessions for about 15 mins a day. Findings converge, that after four sessions, significant health benefits can be found (Pennebaker and Chung, 2007). Cognitive interventions, such as expressive writing tasks, in which participants are asked to write expressively about negative life events, have been reported to improve stress- or emotion regulation and were used in studies with university students (e.g., Sloan et al., 2008; Park et al., 2014; Herbert et al., 2019). Interestingly, also expressive writing about positive events has recently been investigated as a means of positive emotion expression technique in both depressive patients and healthy controls (including university students) (see e.g., Herbert et al., 2019). In the study by Herbert et al. (2019), one session of writing about positive autobiographical events already led to positive effects both on self-reported mood and self-reported bodily symptoms immediately after the writing session (e.g., Herbert et al., 2019). The broaden-and-build theory of positive emotion by Fredrickson (1998) might explain this effect, suggesting that positive writing might lead to a deeper cognitive and emotional processing, which in turn is associated with a heightened attention toward one's needs, creativity and well-being (Fredrickson, 1998; Burton and King, 2004).

Health interventions for university students are mandatory, even more since the Covid-19 pandemic hinders them to carry out their regular exercises. These interventions should adapt to university students' needs as being time-efficient, low-cost, and most suitably directly integrable into the university students' learning routine to strengthen adherence and exercise (Herbert et al., 2020a). On top of that, exercises should be space-saving to be carried out at home with an appropriate level of difficulty (Herbert et al., 2020a). Building upon the previous literature, the present study aimed to find out, whether a program, built upon different physical activity components, e.g., consisting of endurance and strength exercises, but also including mindfulness exercises and also so far not well scientifically examined activities like ballroom dance, could have the same or even an additive effect on mental health of students. Positive effects of dance on stress and depressive symptoms (López-Rodríguez et al., 2017) were already reported in the literature. Dance has also been recommended as a physical activity during the current Covid-19 pandemic by health experts (Hammami et al., 2020), due to its beneficial effects on body and mind. Nevertheless, scientific investigation of effects of ballroom dance on mental health and well-being is underrepresented in the literature. Since ballroom dance steps can be learned and also carried out without a partner, learning dance steps of dances like Discofox or Salsa could be feasible at home, requiring only little space. Furthermore, dancing can be fun (Lima and Vieira, 2007), while at the same time fostering enjoyment and associated adherence (Wankel, 1993; Ryan et al., 1997; Hammami et al., 2020). Similarly, cognitive interventions based on expressive writing, focusing on positive emotion expression, could be particularly useful as an emotion regulation strategy among university students, who have little experience with exercise programs or are anxious to perform on their own. So far, combined interventions, comprising exercise and cognitive interventions, indicate to be promising with regard to variables like perceived stress, depressive symptoms, anxiety or mood (for e.g., see Deckro et al., 2010; de Bruin et al., 2017). However, studies that compare the effects of physical exercise interventions against cognitive interventions like positive expressive writing are still scarce (e.g., see for an overview Herbert et al., 2020b). Such comparisons would allow for an estimation of the advantage and relative effects of physical exercise interventions over cognitive interventions and vice versa among populations at risk of stress-related and mental disorders.

### Aims of the Present Pilot Study: Physical Activity Breaks vs. Cognitive Breaks

The aim of this study was to investigate the effects of a multimodal physical activity intervention on mental health and well-being of university students and to compare the effects with a cognitive intervention, consisting of expressive writing about positive autobiographical events. Crucially, as outlined in the Introduction, the focus was on investigating “activity breaks”, consisting of physical exercises of short duration versus cognitive interventions (writing about positive events), that also allow easy implementation within the daily working schedule of university students. The term “physical activity intervention” was chosen instead of “exercise intervention” because (a) the “activity breaks” comprised different exercise types (described in detail below) and (b) the effects of the two interventions (physical activity versus writing) on physical fitness were investigated with assessment tools, that determined global physical activity behavior (and not necessarily exercise behavior) before and after the interventions. The interventions were directly embedded in the student's learning context. Hence, they were carried out in the classroom and supervised by an exercise professional. The study was carried out before the Covid-19 pandemic across one term (approximately 6 months). Thus, the results are not influenced by the current pandemic situation. Therefore, they can lead to recommendations for beneficial health interventions during the Covid-19 pandemic, based on the regular demands of university students. Moreover, as mentioned above, the focus of our interventions was on low frequency interventions (once a week), whose durations are considered short (~10 mins) and are therefore excellently suited to be performed at home. The different exercises included in the physical activity program require little effort to be carried out on a daily basis in between learning units, irrespective of whether performed at home or in the (virtual) classroom. Concerning the intensity and duration of the investigated physical activity interventions, no physiological adaptations can be expected from the low dose of the interventions according to the previous literature. Thus, in case mental health effects can still be detected, these effects are to be interpreted as due to low dose effects. Moreover, this would suggest, that a mix of low-intensity endurance and muscular strength exercises, mindfulness exercises and exercises related to ballroom dance steps could stimulate the body and the mind by integrating physical and (neuro-)cognitive functions and well-being. Moreover, the physical activity breaks should trigger and increase participants' awareness of generally being more physically active in everyday life.

Similarly, the cognitive intervention of positive expressive writing is expected to have positive effects on university students' mental health, including perceived stress and mood, respectively (e.g., Herbert et al., 2019). Differential effects on mood and stress perception between the two interventions might occur on a weekly basis, but are not primarily expected at T3 (at the end of the interventions). In summary, it is expected, that both interventions will buffer the typical increase of stress and negative mood across the term, associated with the accumulation of cognitive work load from the beginning to the end of term (e.g., Pitt et al., 2018). These questions, concerning the effects of two short interventions on mental health, are addressed in the current study by the assessment of mental health variables, including perceived stress, positive and negative mood, body image, QoL and changes in physical activity or sedentary behavior before and after the participation in the interventions. Of note, it is expected, that these effects hold true for women and men, although in the present study, the final sample consisted of an all-female sample only and comprised a small sample size. This needs to be taken into consideration when talking about the generalizability of the results. However, the two interventions were not developed nor designed for usage of a specific gender, and the aforementioned mental health dimensions were measured via standardized self-report questionnaires (outlined in detail below under section Procedure and study design). This allows comparisons with population norms, despite small sample sizes or a gender bias.

## Materials and Methods

### Participants

Participants were recruited via oral presentation of the study at the beginning of four preselected seminar courses that took place during the winter term 2018/19. The courses were offered by the Department of Applied Emotion and Motivation Psychology at Ulm University. Participation was voluntary and students could opt out whenever they wished. The intervention was introduced and offered by an independent experimenter in four bachelor and master psychology courses. Exclusion criteria for participation were clearly defined, and included an age below 18 years and pre-existing conditions of any physical impairment (e.g., cardiovascular disease or respiratory diseases), hindering the participation in low- to moderate-intensity exercise. Although gender was not an exclusion criterium, due to the gender bias in the courses, only three male students could be recruited as participants and of these, one had missing data. Participants were pre-selected for participation in one of the intervention groups (physical activity versus cognitive intervention group) and care was taken, that the two groups had comparable sample sizes. Two of the university classes were chosen to take part in the (physical) activity intervention group (PAG) and two classes were asked to take part in the cognitive intervention group (CG). Data collection was anonymous and blind for the lecturer of the courses to avoid any bias between the courses and interventions. Among all participants, 10 × 20€ vouchers for *Amazon* were raffled. Alternatively, bachelor students were granted 3.75 h of participation as part of their bachelor student curriculum.

**Figure 1:**
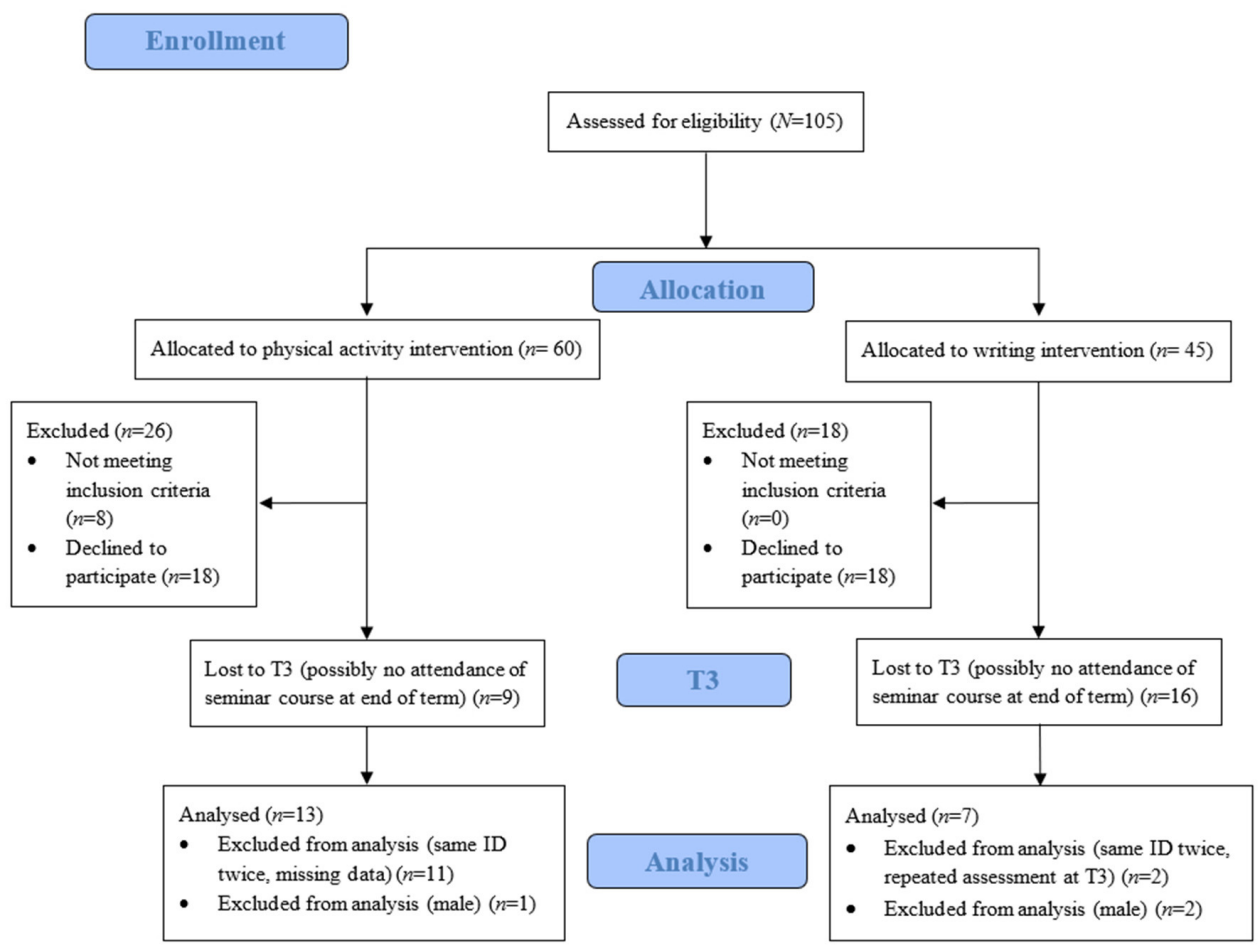
CONSORT diagram depicting dropout numbers, participation numbers and numbers of analysis in the online questionnaire.

Prior to the start of the interventions (T0), participants received a link to an online questionnaire that they filled out from home, 1–7 days before the start of interventions. In addition, the online questionnaire was repeated at four different time points during the intervention period (T1 and T2), at the end of the intervention period (T3) and at follow-up 6 weeks after the last session (T4). Dropout numbers, participation numbers and numbers of analysis can be found in Figure 1. In total, *N* = 56 (PAG: *n* = 32; CG: *n* = 24) participants took part in the first online measurement (baseline, T0). For the per-protocol analysis, only participants having provided data at baseline measurement (T0) and at the end of the term (T3) were taken into account. These two points of time were chosen for the current paper in order to focus on the change in mental health and activity during one semester. Dropout rate from baseline measurement T0 to T3 was 52.17% (PAG: 39.13%; CG: 65.22%). There was no significant relation between group membership and dropout, χ^2^(1) = 3.14, *p* = 0.077, with odds of dropping out having been 0.35 (0.09, 1.32). Finally, an all-female sample was examined. *N* = 20 participants (PAG: *n* = 13; CG: *n* = 7) were analyzed for the comparison between T0 and T3. Sensitivity power analysis in Gpower (Faul et al., 2009) revealed that an effect size of *f* = 0.33 would be required given a Type I error probability of 0.05 and a power of 0.80 with this sample size for between/within comparison.

### Procedure and Study Design

In order to ensure comparable sample sizes, a quasi-experimental design without randomization was chosen. The duration of the individual intervention sessions was 5–10 mins for both groups. The interventions took place every week during the term right at the beginning of the seminar course. The different times of measurement (baseline T0, T1 and T2 during the semester, T3 at the end of term and follow-up T4) were examined to control for differential effects of the two interventions across the intervention period and to determine possible carry-over effects of the intervention from T3 (end of the intervention) to follow-up T4, although T0 to T3 comparisons can be considered to be the most robust comparisons and are the main focus of comparisons reported in section Results of the manuscript. At T0, participants completed a test of selective attention (*d2-R Test for Attention*; Brickenkamp et al., 2010) as paper-pencil version on site at university. They also received a link to the online questionnaire, which they had to fill in at home during the week of T0 (baseline), before the start of the interventions. In 3-week intervals, at T1, T2 and T3, the online questionnaire was repeated and again administered at T4. The T4 follow-up measurement took place online during the semester break, 6 weeks after the last intervention. Participants received one email before each time of measurement, reminding them about filling out the online questionnaires. The online questionnaires were construed with *Unipark* software (Questback GmbH, 2016).

### Measures/Self-Report Questionnaires

All health dimensions of the online questionnaire were assessed via standardized questionnaires. The *Perceived Stress Scale-10* (PSS-10; Cohen et al., 1983) and the *Stress and Coping Inventory* (SCI; Satow, 2012) were used as psychological measures of perceived stress. The PSS-10 (Cohen et al., 1983) is a standardized 10-item scale. Items (e. g. “In the last month, how often have you found that you could not cope with all the things that you had to do?”) are rated on a 5-point Likert-type scale ranging from 0 to 4. Higher scores depict higher perceived stress. The SCI (Satow, 2012) contains ten subscales. A sum score for overall burden with psychological stress was analyzed (21 items; made up of the subscales stress due to uncertainty, stress due to excessive demands and stress due to loss). Every single subscale ranges from 1 to 7 (higher scores equal higher perceived stress). On top of that, the subscale physical and mental stress symptoms (13 items; e. g. “I often suffer from a headache”) was used.

The *World Health Organization-Quality of Life-BREF* (WHOQOL-BREF; The WHOQOL Group, 1998) was administered to measure QoL across certain life domains (physical and psychological health, social relationships and environment). Physical and psychological health domains (13 items, e.g., “How satisfied are you with yourself?”) were analyzed. Scores range from 1 to 5, with higher scores depicting higher QoL.

The *Positive and Negative Affect Schedule* (PANAS; Watson et al., 1988; German version by Krohne et al., 1996) was used to assess state and habitual positive and negative affect. Twenty positive and negative adjectives (e. g. strong or scared) have to be rated on a 5-point Likert-type scale varying from 1 to 5, with higher scores showing higher affective state or trait affect.

The *Global Physical Activity Questionnaire* (GPAQ; Armstrong and Bull, 2006) was used to determine global physical activity levels of the participants prior to the start of the interventions and at the end of interventions. The GPAQ (Armstrong and Bull, 2006) asks for regular physical activity of moderate and vigorous intensity at work, during travel to and from places and activity as recreational activities. It also measures sitting time (sedentary time) in hours and minutes per week. Physical activity per day can be obtained including the three domains at work, travel to and from places and recreational activity.

To determine risk of depression, the *Beck Depression Inventory-II* (BDI-II; Beck et al., 1996; German version by Hautzinger et al., 2006) was administered. It consists of 21 items (e.g., “I am so sad or unhappy that I can't stand it”) and is measured on a Likert-type scale with a range from 0 to 4 (higher scores corresponding to higher depressive symptomatology).

The *Eating Disorder Inventory-2* (EDI-2; Garner, 1991; German version by Thiel et al., 1997) measures cognitive and behavioral dimensions of eating disorders. It comprises eleven subscales. Only the subscale Body Dissatisfaction was used (8 items; e. g., “I think that my thighs are too large”). Higher scores equal higher body dissatisfaction.

Moreover, the *Physical Activity Readiness Questionnaire* (PAR-Q; Shepard, 1988) was included for the assessment of possible health restrictions, preventing participation in the physical activity program in the physical activity group. Seven possible health restrictions were queried. One of these items (dizziness) was left out because the discriminatory power is doubted. In case one of the six remaining items was answered with yes (e.g., “Do you feel pain in your chest when you perform physical activity?”), the participant was excluded directly from the questionnaire. The PAR-Q (Shepard, 1988) was only used for the PAG because the CG did not carry out any physical activity.

The *Balanced Inventory of Desirable Responding* (BIDR; Paulhus, 1988; German version by Musch et al., 2002) was used in order to control for the tendency of answering with social responding. Only the subscale Impression management (10 items; e.g., “I never swear”) was administered. It can be rated on a scale ranging from 1 to 7, with lower scores depicting a higher social desirability tendency.

The *Reasons for Exercise Inventory* (REI; Silberstein et al., 1988) was administered to assess participants' previous exercise motivation and reasons for exercising. It is a 24-item scale measuring seven different reasons to work out (Mood, Fitness, Health, Enjoyment, Weight Control, Tone, Attractiveness; sample item: “To have fun”). Participants rate these dimensions on a scale from 1 to 7. Higher scores support the consent on a particular reason for exercise. The results of the REI can be found in the Supplementary Material of this manuscript.

Selective attention was measured directly on-site during courses via the *d2-R Test for Attention* (Brickenkamp et al., 2010) as paper-pencil version. The d2-R is a standardized cognitive measurement. Certain letters on several rows have to be crossed out, while others have to be left out. Speed and accuracy are most important. The factor ability to concentrate was used for the current study. The results of the d2-R (Brickenkamp et al., 2010) can be found in the Supplementary Material of this manuscript.

The PANAS state (Watson et al., 1988; Krohne et al., 1996) as well as the GPAQ (Armstrong and Bull, 2006) and the PSS-10 (Cohen et al., 1983) were assessed at all measurement times to control for differential intervention effects on mood and stress across the duration of the two interventions (physical activity versus positive expressive writing). The self-report questionnaire at T2 additionally contained the WHOQOL-BREF (The WHOQOL Group, 1998) for QoL. Primary and secondary variables were again assessed completely at T3 and at follow-up (T4). The d2-R (Brickenkamp et al., 2010) was applied at all times of measurement during the term (at T0, T1, T2 and T3). Results concerning the d2-R (Brickenkamp et al., 2010) and reasons for exercise (REI; Silberstein et al., 1988) can be examined in the Supplemental Material for a subset of the participants.

The cognitive intervention was also analyzed descriptively for its content, i.e. number of words, and the semantic and emotional categories they belong to were analyzed by using the Linguistic Inquiry Word Count (LIWC; Pennebaker et al., 2001; http://liwc.wpengine.com) tool. The LIWC (Pennebaker et al., 2001) is a quantitative text analysis program, counting and categorizing words by predetermined content (e.g., emotional content, social contents, function words, self-referential content words, etc.).

### Interventions

#### (Physical) Activity Intervention

**Figure 2:**
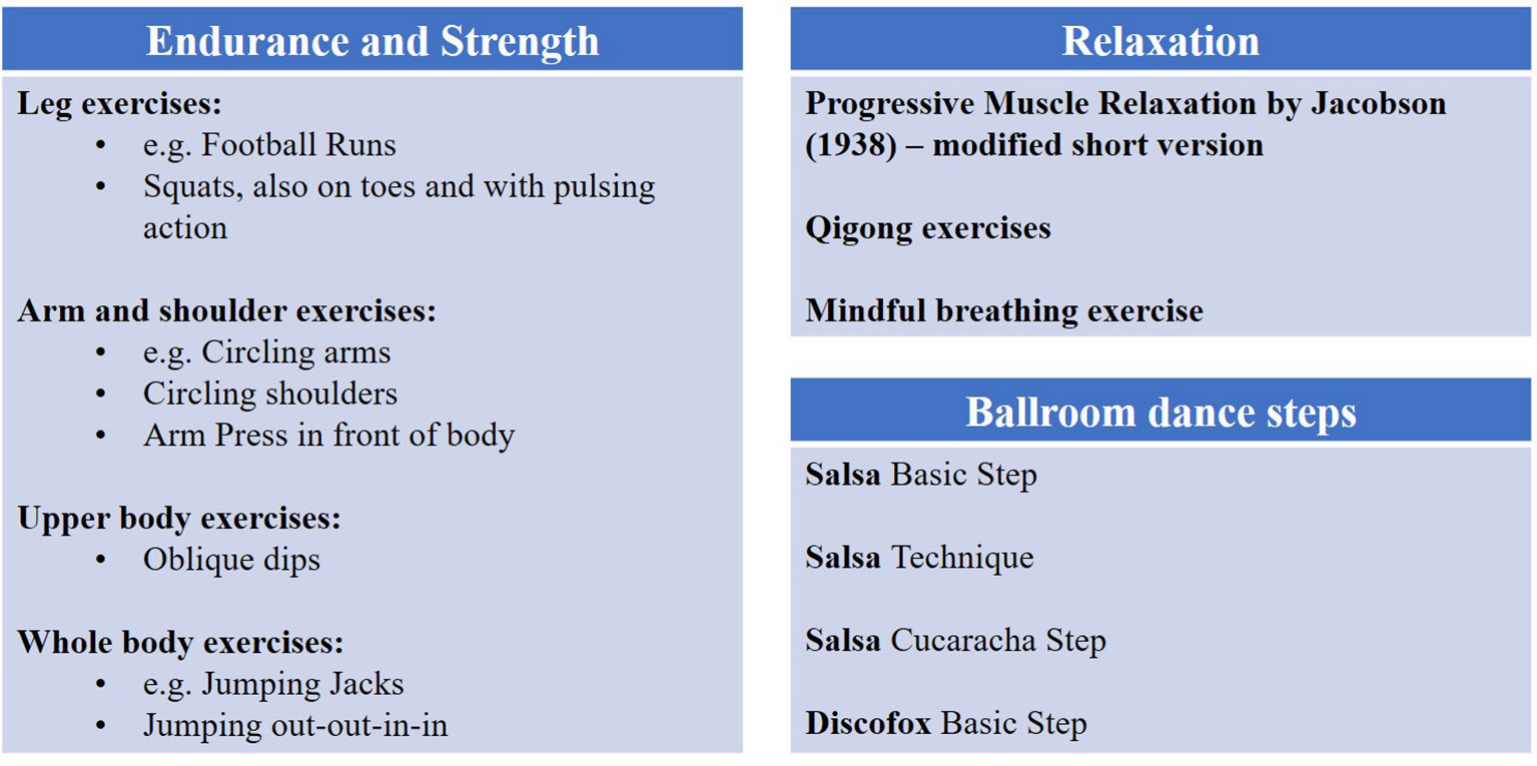
Selection of exercises as part of the physical activity program. Muscular and endurance exercises were based on and adapted of the fitness program *Blogilates* by Cassey Ho (www.blogilates.com), inspired by high-intensity interval training with each exercise being carried out for 1–2 mins with short breaks of 10–15 s in between. Relaxation sessions consisted of a modified version of Progressive Muscle Relaxation (PMR; Jacobson, 1938), qigong exercises and a mindful breathing exercise developed by Department of Applied Emotion and Motivation Psychology of Ulm University. Ballroom dance steps were taught by a certified dance teacher according to the guidelines by the *Allgemeiner Deutscher Tanzlehrerverband e.V. (ADTV)*. The steps were carried out without a partner. Progressive Muscle Relaxation-modified short version by Department of Applied Emotion and Motivation Psychology of Ulm University, originally by Jacobson (1938).

The physical activity group received weekly physical activity breaks provided by the authors. The physical activity breaks followed standardized exercise protocols and physical activity guidelines (see Figure 2 for an overview). As shown in Figure 2, the individual exercises of the physical activity break comprised different components: aerobic endurance and muscular strength exercises, relaxation and dance steps (see Figure 2 for example exercises). All of the exercises are suitable to be done with limited space, at home or during e-learning, either in a standing position or seated on a chair. Each component was carried out 3 weeks in a row in the specified order. The exercises were guided by an exercise model, hence the possibility for correction and motivation enhancement was given in order to improve adherence. Participants were instructed to carry out the exercises according to the exercise model, who always stood in the front of the room, facing the students. The exercise model gave instructions for each exercise, e.g., in terms of proper posture and execution.

#### Cognitive Intervention

The cognitive intervention also followed standardized protocols. It comprised a positive expressive writing task, developed by the corresponding author and used in previous studies (e.g., Herbert et al., 2019). The instructions of the positive expressive writing task were adapted from the original expressive writing protocol, provided by Pennebaker and colleagues (e.g., Pennebaker, 2018). Accordingly, participants were asked to write about own feelings without focusing on spelling or grammar. However, in contrast to the typical expressive writing conditions, asking to write about traumatizing negative live events across repeated sessions, in the present study, the participants were asked to expressively write about positive autobiographical life events only. They were free to write about any personal positive experiences that came to mind. The writing task was carried out as paper-pencil task. The cognitive intervention group was matched to the physical activity group (e.g., with regard to duration of the sessions of the intervention, the setting and guidance by an instructor).

### Data Analysis and Statistics

The statistical analysis was carried out with the statistics program *RStudio* (Version 1.2.5033; R Studio Team, 2019). In order to find possible differences in dropout rate, Pearson's chi square test was used. Possible group differences concerning continuous variables, i.e. age, depression, body dissatisfaction, etc., were examined via linear regression with the factor *group*. In case the assumptions of linear regression were violated, a bootstrapping procedure following Field et al. (2012, pp. 298–301) was used. For the main analyses, data were filtered in order to leave only participants with T0 and T3 measurements, hence following per-protocol analysis. Group comparisons over time (*group* × *time* interactions) were calculated via linear mixed-effects models with the R package *nlme* (Pinheiro et al., 2019). *Group* and *time* were dummy-coded. Variables age, depression and degree were included in the models if there was a significant difference between groups in order to control for these differences. Effect size *r* was chosen, as recommended by Field et al. (2012, pp. 640–641). In case of violation of assumptions for the models, a two-way between-within subjects analysis of variance (ANOVA) of trimmed means (20%), as developed by Wilcox (2017) was carried out, included in the *WRS2* package (Mair and Wilcox, 2020). Unfortunately, the *bwtrim* function of this package only computes a two-way between-within subjects ANOVA on trimmed means designed for one between-subjects and one within-subjects variable. Hence, the inclusion of further variables, e.g., depression or age as control variables, is not possible with this package. The *p*-value for significance was set to *p* ≤ 0.05.

## Results

### Statistics of the Two Groups Before the Start of the Intervention (T0)

As can be seen in Table 1, the cognitive intervention group had a higher mean age (*M* = 24.14, *SD* = 0.90) than the physical activity group (*M* = 22.39, *SD* = 2.02). This difference was significant, *B* = −1.76, *SE*_*B*_ = 0.81, *t*(18) = −2.17, *p* < 0.05. Pursued university degree differed between the two groups, with the PAG having consisted of more bachelor than master students than the CG (see Table 1). In the cognitive intervention group, the majority of students pursued a master degree. The distribution was significantly different between groups, χ^2^(1) = 5.80, *p* < 0.05. Across groups, the majority of students (35.00%) reported to spend 5–10 h per week at university to perform classes, courses and lectures, followed by 30.00% of students, who reported to spend 10–15 h per week at university for learning activities (teaching). 25.00% reported to attend lectures for 15–20 h per week, while 10.00% had a higher attendance time of more than 25 h per week. None of the student participants spent < 5 h per week at university.

**Table 1:** Baseline self-report data of participants in dependence of group with means and standard deviations.

	**Group**	
	**Physical activity group**	**Cognitive intervention group**
Sample size	13	7
Mean age	22.39 (2.02)	24.14 (0.90)
Pursued university degree	7 Bachelor degree 6 Master degree	0 Bachelor degree 7 Master degree
Mean university activity per week at home (in hours)	18.31 (9.80)	16.00 (9.88)
BDI-II (depression)	3.85 (3.65)	10.14 (7.63)
EDI-2 (body dissatisfaction)	29.77 (7.97)	26.86 (7.24)
GPAQ (total physical activity per day in minutes)	62.86 (48.99)	137.96 (148.64)
GPAQ (sedentary time per day in minutes)	574.62 (197.43)	638.57 (346.72)

*Data from online-questionnaire. University activity encompasses attendance time and activities at home. The following questionnaires and scales were used: Beck Depression Inventory-II (BDI-II; Beck et al., 1996; German version by Hautzinger et al., 2006), Eating Disorder Inventory-2 (EDI-2; Garner, 1991; German version by Thiel et al., 1997), Global Physical Activity Questionnaire (GPAQ; Armstrong and Bull, 2006)*.

At T0 (baseline measurement), the cognitive intervention group had a higher mean depression score compared to the physical activity group on the Beck Depression Inventory-II (BDI-II; Beck et al., 1996; German version by Hautzinger et al., 2006). There was a significant difference between groups, 95% CI [−13.68, −1.60]. According to the cut-off scores of the BDI, the PAG can be categorized as a non-depressive sample (*M* = 3.85, *SD* = 3.65, *range* = 0–11), while the cognitive intervention group had a mean score classified as minimal depression (*M* = 10.14, *SD* = 7.63, *range* = 3–23). Body dissatisfaction, as indicator for eating disorders in the Eating Disorder Inventory-2 (EDI-2; Garner, 1991; German version by Thiel et al., 1997), at baseline measurement (see Table 1) can be categorized as unobtrusive (score below 35.6 as cut-off score in a validation sample of anorexia nervosa patients; Paul and Thiel, 2005). Although the PAG had higher body dissatisfaction scores, there was no significant difference between groups, *B* = 2.91, *SE*_*B*_ = 3.63, *t*(18) = 0.80, *p* = 0.432. At T0 (baseline), 15.00% of participants did not meet the recommendations of physical activity for health as stated by the World Health Organization (World Health Organization, 2010). Physical activity per day, measured with the Global Physical Activity Questionnaire (GPAQ; Armstrong and Bull, 2006), was 89.14 mins on average (*SD* = 99.21). The CG reported approximately double the amount of physical activity compared to the PAG at T0, although there was no significant difference between groups, 95% CI [–233.34, 6.19]. Sedentary time per day was 597.00 mins on average (=9.95 h per day; *SD* = 252.11 mins). Both groups were comparable in sedentary time, 95% CI [−470.94, 125.88]. Participants were asked about the number of sessions they attended during the last 3 weeks. On average, they attended 1.80 sessions (*SD* = 0.70). Number of participation was equal among both groups (PAG: *M* = 1.85, *SD* = 0.80; CG: *M* = 1.71, *SD* = 0.49), 95% CI [−0.40, 0.70].

### Comparisons Between Intervention Groups Over Time (T0 vs. T3)

Descriptive statistics for times of measurement T0 with T3 are depicted in Table 2. Perceived stress, measured via the PSS (Cohen et al., 1983), was higher for the cognitive intervention group than for the physical activity group independent from time of measurement, with a significant main effect for *group, Q* = 6.77, *p* < 0.05. As expected (due to assessment phase at the end of term), stress levels were generally marginally higher at T3 compared to T0, although not significantly, *Q* = 1.95, *p* = 0.203. The *group* × *time* interaction was not significant, *Q* = 0.04, *p* = 0.841. The same as in the PSS (Cohen et al., 1983) for the factor *group* applied for overall stress burden and physical and mental stress symptoms of the SCI (Satow, 2012): The cognitive intervention group showed higher stress levels, although not significantly, overall stress burden: *Q* = 3.26, *p* = 0.127, stress symptoms: *Q* = 1.63, *p* = 0.258. Perceived stress was marginally lower at T3 compared to T0 for overall stress burden, *Q* = 1.24, *p* = 0.313, and stress symptoms decreased for the physical activity group, but increased for the cognitive intervention group, *Q* = 0.01, *p* = 0.934. The *group* × *time* interaction for both subscales was not significant, overall stress burden: *Q* = 0.30, *p* = 0.607, physical and mental stress symptoms: *Q* = 0.09, *p* = 0.774. Both main effects of *group* and *time* for positive state affect were not significant, *group: Q* = 0.25, *p* = 0.640, *time: Q* = 0.41, *p* = 0.543. This was also found for the interaction effect *group* × *time, Q* = 0.19, *p* = 0.673. Overall, the cognitive intervention group reported a higher negative state affect compared to the physical activity group, although this main effect of *group* was not significant, *Q* = 3.98, *p* = 0.108. Both the physical activity group and the cognitive intervention group reported a decrease in negative affect over time, however with the main effect of *time* having been not significant, *Q* = 0.45, *p* = 0.536. There was no significant interaction effect, *Q* = 0.04, *p* = 0.841.

**Table 2:** Mean scores and standard deviations (SDs in brackets) of scales in dependence of time of measurement (online questionnaire: physical activity group with *n* = 13, cognitive intervention group with *n* = 7).

**Questionnaires (range)**	**Time of measurement**			
	**T0 (Baseline)**		**T3**	
	**Physical activity group**	**Cognitive intervention group**	**Physical activity group**	**Cognitive intervention group**
PSS-10 Perceived stress (0–40)	8.08 (3.20)	11.29 (4.39)	8.85 (3.21)	12.14 (2.91)
SCI				
Overall stress burden (21–147)	40.23 (11.27)	52.29 (19.33)	35.46 (7.83)	50.43 (15.14)
Physical and mental stress symptoms (1–4)	1.59 (0.35)	1.89 (0.62)	1.52 (0.21)	1.99 (0.79)
PANAS				
Positive state (1–5)	2.53 (0.84)	2.57 (0.75)	2.54 (0.57)	2.33 (0.36)
Negative state (1–5)	1.31 (0.36)	1.99 (0.90)	1.23 (0.25)	1.79 (0.46)
GPAQ				
Total physical activity per day in minutes	62.86 (48.99)	137.96 (148.64)	63.85 (44.92)	51.33 (42.98)
Sedentary time per day in minutes	574.62 (197.43)	638.57 (346.72)	588.46 (155.66)	608.57 (123.35)
WHOQOL-BREF				
Physical domain (4–20)	17.46 (0.88)	16.00 (2.08)	17.08 (1.04)	15.00 (1.83)
Psychological domain (4–20)	15.62 (1.33)	15.00 (3.22)	15.69 (1.97)	14.57 (2.30)

*T3 measurement was taken at the end of semester. The following questionnaires/tests (and subscales) were used: Perceived Stress Scale-10 (PSS-10; Cohen et al., 1983), Stress and Coping Inventory (SCI; Satow, 2012), Positive and Negative Affect Scale (PANAS; German version by Watson et al., 1988; Krohne et al., 1996), Global Physical Activity Questionnaire (GPAQ; Armstrong and Bull, 2006), and World Health Organization-Quality of Life-BREF (WHOQOL-BREF; The WHOQOL Group, 1998)*.

**Figure 3:**
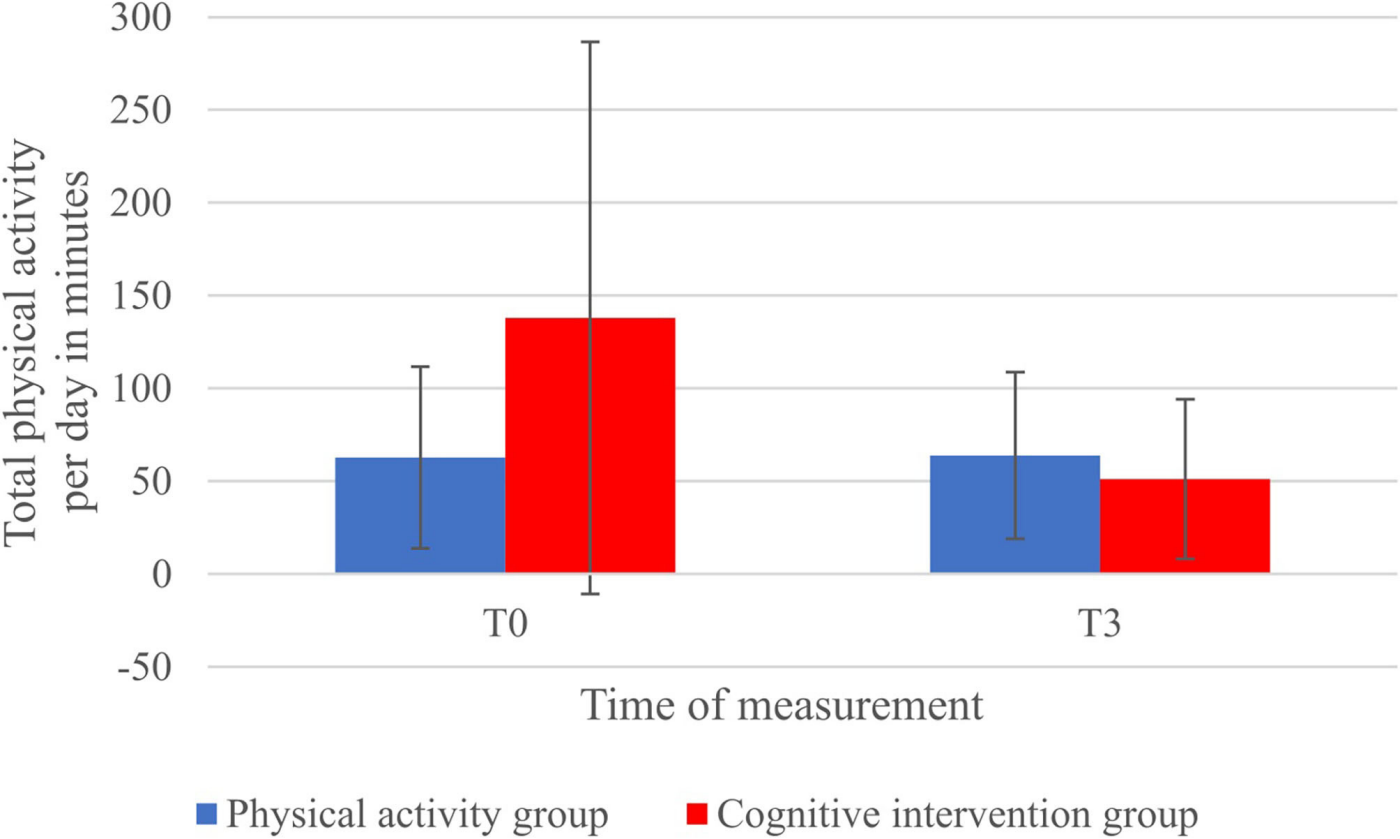
Changes in mean total physical activity per day in minutes as measured by the *Global Physical Activity Questionnaire* (GPAQ; Armstrong and Bull, 2006) from T0 (baseline measurement) to T3 (end of semester) in dependence of group. Figure shows significant *group* × *time* interaction. Error bars depict standard deviations. *p* < 0.05.

Concerning physical activity per day, as can be seen in Figure 3, the cognitive intervention group showed a higher activity score at T0 than the physical activity group. While the activity score of the PAG increased slightly, the CG reported a much lower score compared to T0 and also lower compared to the activity group. This interaction *group* × *time* was significant, *B* = 86.18, *SE*_*B*_ = 35.23, *t*(17) = 2.45, *p* < 0.05, *r* = 0.51. Factors *group, B* = −92.65, *SE*_*B*_ = 42.90, *t*(16) = −2.16, *p* < 0.05, *r* = 0.48, and *time, B* = −86.63, *SE*_*B*_ = 28.39, *t*(17) = −3.05, *p* < 0.01, *r* = 0.60, also showed a significant effect. Differences between groups over time were especially seen for physical activity related to travel to and from places and vigorous, as well as moderate recreational activity per day (in minutes), as can be seen in Table 3. While the physical activity group showed no changes in physical activity while traveling or working out recreationally, traveling and especially vigorous recreational physical activity decreased for the cognitive intervention group. Moderate-intensity recreational activity increased for the CG, although unrelated to the high decrease in vigorous-intensity recreational activity for this group. All group and time effects for the subscales were not significant, all *p*s > 0.05. The same was found for the interaction effects of the subscales (vigorous work activity per day: *B* = −0.87, *SE*_*B*_ = 13.06, *t*(17) = −0.07, *p* = 0.947; moderate work activity per day: *Q* = 0.47, *p* = 0.321; travel to and from places: *B* = 19.14, *SE*_*B*_ = 9.81, *t*(17) = 1.95, *p* = 0.068; vigorous recreational activity: *B* = 105.86, *SE*_*B*_ = 65.39, *t*(17) = 1.62, *p* = 0.124; moderate recreational activity: *Q* = 0.45, *p* = 0.533).

**Table 3:** Mean scores and standard deviations (SDs in brackets) of Global Physical Activity Questionnaire subscales in dependence of time of measurement and groups.

**Subscale**	**Time of measurement**			
	**T0 (Baseline)**		**T3**	
	**Physical activity group**	**Cognitive intervention group**	**Physical activity group**	**Cognitive intervention group**
GPAQ				
Vigorous work activity per day in minutes	1.15 (4.16)	0.00 (0.00)	9.23 (33.28)	8.57 (22.68)
Moderate work activity per day in minutes	16.92 (28.69)	34.29 (58.56)	20.00 (30.21)	24.29 (43.92)
Travel to and from places per day in minutes	29.62 (22.86)	47.14 (41.92)	29.23 (28.93)	27.86 (19.55)
Vigorous recreational activity per day in minutes	53.46 (52.26)	115.71 (219.38)	58.85 (47.62)	12.86 (34.02)
Moderate recreational activity per day in minutes	42.69 (38.71)	22.86 (33.02)	46.54 (54.37)	45.71 (55.03)

*T3 measurement was taken at the end of semester. Global Physical Activity Questionnaire (GPAQ; Armstrong and Bull, 2006)*.

Sedentary time was higher for the cognitive intervention group compared to the physical activity group. It increased for the PAG from T0 to T3, but decreased for the CG from T0 to T3. Both main effects were not significant, *group: Q* = 0.01, *p* = 0.911, *time: Q* = 1.26, *p* = 0.306, as was the interaction effect, *Q* = 0.46, *p* = 0.524. The physical activity group showed a higher QoL score for the physical domain and the psychological domain of the WHOQOL-BREF (The WHOQOL Group, 1998) compared to the cognitive intervention group, although this group effect was not significant, physical domain: *Q* = 5.54, *p* = 0.070, psychological domain: *Q* = 0.52, *p* = 0.503. The physical score decreased for both groups from T0 to T3, *Q* = 6.58, *p* < 0.05. The psychological score remained unchanged for the activity group and decreased marginally for the cognitive intervention group, *Q* = 0.35, *p* = 0.573. There was no significant interaction effect of *group* × *time* for both domains, physical domain: *Q* = 3.11, *p* = 0.118, psychological domain: *Q* = 0.35, *p* = 0.573.

### Word Use: Descriptive Data Analytics as Manipulation Check

As expected, positive writing was characterized by a higher use of positive emotion words (*M* = 6.30) than negative emotion words (*M* = 0.80) across the weekly sessions. Use of discrete negative emotion words related to anger (*M* = 0), sadness (*M* = 0.53) or fear (*M* = 0.13%) was negligible and almost not present. Authenticity (*M* = 74) of the writing as well as the tone (*M* = 83.57) of the writing were well pronounced, suggesting that writings were emotional, personal and self-revealing in all sessions. A high number of words was categorized into the “social” category of the LIWC dictionary (Pennebaker et al., 2001) and content of writing seemed to be equally distributed about work (*M* = 2.02) and leisure (*M* = 2.64), followed by home (*M* = 1.23), while categories related to death (*M* = 0.02) and money (*M* = 0.30) were underrepresented. Similarly, writing was characterized by present tense (*M* = 7.65) rather than past (*M* = 4.85) or future (*M* = 0.51) tense.

## Discussion

The purpose of the study was to compare the effects of two interventions, consisting of short activity breaks, one of them being a physical activity break and the other one being a cognitive intervention of positive expressive writing. These were compared with regard to their potential to alleviate mental health (perceived stress, positive and negative affect, QoL) and to improve habitual physical activity among university students across the university term. The physical activity intervention comprised a multimodal physical activity break of a duration of 5–10 mins, including endurance, strength, relaxation and dance exercises. The cognitive intervention consisted of an expressive writing task about positive autobiographical events. Both interventions were developed by the authors and followed a standardized in-house protocol. The interventions were carried out and guided once a week at the beginning of a weekly class for one term. Self-report measures, assessing perceived stress, mood/affect, QoL, and habitual physical activity behavior before, during and after the interventions (at the end of one semester), were administered. The interaction effect of *group* × *time* on self-reported perceived stress, mood/affect and QoL was not significant. Concerning physical activity, there was a significant interaction effect of *group* × *time* with a decrease in activity level for the cognitive intervention group, while the physical activity group had a stable unchanged activity level from the beginning to the end of one semester.

These findings point toward differential and specific effects of the interventions. Compared to the cognitive intervention, the physical activity intervention may have helped to better become aware of physical activity behavior and thus, to keep up one's physical activity level across the time course of the term. This is also supported by the fact that the cognitive intervention group started with a higher physical activity level than the physical activity group at T0 (baseline). Nevertheless, physical activity dropped from T0 to T3. Whether this is biased by a negative effect of the cognitive intervention on physical activity of the students, cannot be confirmed in the present study, but should definitely be exploited in further studies. Likewise, whether the physical activity intervention served as a buffer for possible inactivity effects during the assessment period, should be investigated in future studies, as this would support the assumption that even a single bout (here session) of short-term exercises can temporarily change physical activity. Alternatively, a lower time of physical activity per day might be generally maintained, even without any intervention. This can only be explored by including a passive control group. Therefore, another study comparing physical activity and cognitive interventions with a passive control group should be conducted.

Positive effects of physical activity interventions on physical activity levels are mainly found in classroom-based studies with school children (e.g., Murtagh et al., 2013; Drummy et al., 2016). These findings cannot be generalized to university students, therefore, further studies, examining effects of physical activity breaks on physical activity levels in university and college students, are needed. The specific effects of work, travel or recreational activity in this study were not significant, while the overall physical activity interaction between *time* and *group* was. Still, the descriptive analysis pointed toward changes in traveling as well as vigorous-intensity recreational activity. Whether there is an association between these changes and the kind of intervention, needs to be examined further. It would be interesting to know, whether the activity break was also carried out during leisure time as well, or whether it could lead to the maintenance of carrying out one's own preferable type of exercise. In the current study, physical activity was measured subjectively via questionnaire. A good objective measure for future studies could be accelerometry or heart rate monitoring (Strath et al., 2013).

The results and intervention design of the present pilot study can be seen as a starting point for recommendations for the inclusion of physical and cognitive activity breaks in the learning context of university students during the current Covid-19 pandemic. As already mentioned, the exercises chosen are based on important exercise dimensions, fostering endurance and strength, relaxation and ballroom. All exercises are provided at an intensity, duration, frequency and expertise level that allows their engagement without much practice. Our interventions might even be extended to different settings outside the university context, e.g., office buildings or home office, or to different target groups, e.g., older people. Since many people suffer from a rather inactive lifestyle, even more so because of Covid-19, and stress also being on the rise (Techniker Krankenkasse, 2016), physical activity and stress regulatory programs are urgently needed, even for target groups, that do not suffer from any health burdens at the start of the Covid-19 pandemic. During times like these, where social distancing is so far one of the best ways to combat the Covid-19 pandemic, we are in need of suitable, short space- and time-efficient activity programs (e.g., Lippi et al., 2020; Woods et al., 2020), as provided in the present pilot study. Furthermore, positive expressive writing does not cause the unwanted side effects of traditional expressive writing about negative events, the latter leading to an increase in negative mood after the first sessions, before unfolding positive effects earliest after the fourth session (Pennebaker and Chung, 2007). Descriptive analysis of word use of the texts written by participants of the cognitive intervention group showed, that on average, use of positive words was higher than use of negative words. This suggests a positivity bias (e.g., Herbert et al., 2019). Theoretically, a positivity bias can act as buffer against negative mood and mental ill health, as suggested by broaden-and-build theory (Fredrickson, 1998). On top of that, positive expressive writing might be a suitable activity for people that are rather not interested in exercising.

Regarding the current Covid-19 pandemic, students can surely benefit from the physical, as well as the cognitive intervention, if both interventions will be provided as online interventions. With regard to physical activity levels, smartphone physical activity interventions showed to be promising in increasing activity during leisure time (Feter et al., 2019). Since the type of exercise as well as its intensity and pre-existing health burdens of the exerciser play a significant role with respect to which protective effects an exercise intervention can exert on mental health (Gronwald et al., 2018), the exercises included in the current intervention should be rated by experts for their effectivity beforehand (e.g., see recommendations by Herbert et al., 2020a).

Physical activity and exercise interventions can be regarded as promising with high effect sizes, concerning the decrease of depressive symptoms and anxiety disorders (Huang et al., 2018). We assessed mental health at T0, i.e. prior to the start of the intervention. Assessment included, amongst others, risk for depression and eating disorders at T0 (baseline before the start of the interventions). The rate of mental diseases like depression and anxiety in university and college students is high (Huang et al., 2018), specifically now during Covid-19 (Islam et al., 2020). Furthermore, risk of eating disorders and body dissatisfaction, an important predictor of eating disorders, seem to be prevalent and common in academic samples of adults (e.g., Eisenberg et al., 2011; Radwan et al., 2019). Interestingly, in the present small sample, depression scores and the risk for eating disorders were considered low, compared to standardized norms or cut-off scores. Nevertheless, depression and risk for eating disorders could have been higher during the term and at the end of term. In total, no significant interaction effects of *group* and *time* were found for university students' perceived stress, self-reported positive and negative state affect, the reported sedentary time, and psychological and physical dimensions of QoL from T0 (baseline before the intervention) to T3 (at the end of one term). This suggests, that both interventions had no differential effects on these variables. Interestingly, this was the case, although at T0, the two intervention groups differed in age and mean score of depressive symptoms, as assessed with the BDI-II (Beck et al., 1996; German version by Hautzinger et al., 2006). The mean scores of the student samples, participating in the two interventions, were comparable on all other dimensions at baseline (T0), including global physical activity behavior.

Although mental health did not increase due to the interventions in this study, other studies point toward positive effects of both kind of interventions on mood and perceived stress (e.g., Dolbier and Rush, 2012; Pennebaker, 2018; Kramer, 2020). With regard to perceived stress, it could be presumed, that an increase would have taken place from T0 to T3, because of T3 having been during examination period at the end of term. Since there was no significant effect of *time*, both interventions might have successfully buffered the negative effects of exams on university students' mental and physical health. Given that a group receiving no intervention was not included in the study, to allow every student interested in the study to benefit from an intervention, we cannot make any inferences about usual changes in mental health. Whether a passive control group without intervention showed an increase in perceived stress toward the end compared to both active interventions remains unknown. This should definitely be evaluated in future studies. Still, we refer to the literature and the teaching curricula, clearly documenting an increase of academic stressors within the time period of T3. Hence, the present results concerning perceived stress and also affect and QoL could have emerged because of a heightened stress level, lower positive and higher negative affect and lower QoL during examination period. Since there were no direct measurements taken right before and after each session, short lived immediate effects on students' mood and well-being were not captured. This would be an interesting research topic for future studies.

It should be noted that the exercises we offered can be characterized as being of low dose, i.e., possibly too low to elicit any physiological training effects across the six weeks of the interventions. Nevertheless, the exercises of our physical activity intervention could be expected to have good effects for previously sedentary students. Studies support the notion that even short breaks in sedentary behavior can have positive effects on the body (e.g., might help to control adiposity; Chastin et al., 2015) and on mental health (e.g., lower the odds of depression and anxiety symptoms; Hallgren et al., 2020). Therefore, our physical activity break might be appropriate to use as such a break regarding sedentary behavior. The exercises can be carried out without the use of much space or time and are therefore suitable for people with a high sedentary time such as university students (Castro et al., 2020). Overall sedentary time did not change in the current study, but interruptions in sedentary time were not assessed. This should be done in future studies.

A few limitations of the current study need to be addressed. Concerning the very small final sample size of our pilot study, *post-hoc* power analysis (Gpower, Faul et al., 2009) recommends a sample size of *N* = 98 in order to get a medium effect size with a Type I error probability of 0.05 and a power of 0.80 (as recommended by Cohen, 1988). Therefore, a higher sample size in order to increase power is needed. Of note, as mentioned under “Aim of this pilot study”, the final sample was an all-female sample. For this reason, the interpretation of results, as well as the use of interventions, might be reliable for female university students. Future studies should include male students and students of different courses of study to increase generalizability. However, the exercises included in the physical activity break, as well as the cognitive intervention, are based on basic skills that might not vary dramatically across gender. In this study randomization was not carried out in order to prevent an unbalanced design due to different sizes of lectures. However, this limits internal validity. Therefore, future studies should use an experimental design with randomization of students.

All in all, the exercises included in the physical activity break as well as the cognitive intervention can easily be integrated into the university context, e.g., by offering them either during online lectures or on-site at university, which might also lower the feeling of loneliness and isolation, if carried out together. Alternatively, the interventions can be offered app- or web-based, to be carried out asynchronously at home by oneself. The approach of the present interventions, even their combination (physical activity and positive expressive writing), could produce good adherence, because of their multimodality and flexibility and the possibility to be included easily as breaks in between learning and working hours (Hammami et al., 2020). In summary, extending existing research to provide programs to fight sedentarism, physical inactivity and mental ill health during the current Covid-19 pandemic, the developed and explored activity breaks used in this pilot study might be seen as fruitful endeavors.

## Data Availability Statement

The data supporting the conclusions of this article will be made available by the corresponding author, without undue reservation, to any qualified researcher. Due to the informed consent form in which the possibility of raw data being published online was not explicitly stated, only group-level data, as it is provided in this article, can be made accessible upon request.

## Ethics Statement

Participants had to give written informed consent to confirm the exclusion criteria and were debriefed about the purpose of the study, following ethical guidelines and study protocol standards, used in the department's previous physical activity and cognitive intervention studies, which had been approved by the local ethics committee of Ulm University (https://www.uni-ulm.de/einrichtungen/ethikkommission-der-universitaet-ulm/). Therefore, for this study, no ethics approval was submitted before the start of the study.

## Author Contributions

VM and CH (contributed equally): conceptualization, writing—original draft, and review and editing. VM and CH: data curation, methodology, validation, and investigation. VM: formal analysis and visualization. CH: resources, supervision, project administration, and funding. All authors have approved the final version of the manuscript. Both authors contributed equally to the parts with equal contribution.

## Conflict of Interest

The authors declare that the research was conducted in the absence of any commercial or financial relationships that could be construed as a potential conflict of interest.

## Publisher's Note

All claims expressed in this article are solely those of the authors and do not necessarily represent those of their affiliated organizations, or those of the publisher, the editors and the reviewers. Any product that may be evaluated in this article, or claim that may be made by its manufacturer, is not guaranteed or endorsed by the publisher.

## References

[B1] American College of Sports Medicine (2000). ACSM's Guidelines for Exercise Testing and Prescription, 6th ed. Philadelphia, PA: Lippincott, Williams and Wilkins.

[B2] Armstrong T. Bull F. (2006). Development of the World Health Organization Global Physical Activity Questionnaire (GPAQ). J. Public Health (Oxf). 14, 66–70. 10.1007/s10389-006-0024-x

[B3] Baghurst T. Kelley B. C. (2014). An examination of stress in college students over the course of a semester. Health Promot. Pract. 15, 438–447. 10.1177/1524839913510316 24231633

[B4] Baikie K. A. Geerligs L. Wilhelm K. (2012). Expressive writing and positive writing for participants with mood disorders: an online randomized controlled trial. J. Affect. Disord. 136, 310–319. 10.1016/j.jad.2011.11.032 22209127

[B5] Bassett-Gunter R. McEwan D. Kamarhie A. (2017). Physical activity and body image among men and boys: a meta-analysis. Body Image 22, 114–128. 10.1016/j.bodyim.2017.06.007 28756298

[B6] Beck A. T. Steer R. A. Brown G. K. (1996). Beck Depression Inventory–II (BDI–II): manual, 2nd ed. San Antonio, TX: Harcourt Assessment Inc.

[B7] Blair S. N. Cheng Y. Holder J. S. (2001). Is physical activity or physical fitness more important in defining health benefits? Med. Sci. Sports Exerc. 33, 379–399. 10.1097/00005768-200106001-00007 11427763

[B8] Brickenkamp R. Schmidt-Atzert L. Liepmann D. (2010). Attention and Concentration Test d2-Revised Version. Göttingen: Hogrefe Verlag.

[B9] Burton C. M. King L. A. (2004). The health benefits of writing about intensely positive experiences. J. Res. Pers. 38, 150–163. 10.1016/S0092-6566(03)00058-8

[B10] Campbell A. Hausenblas H. A. (2009). Effects of exercise interventions on body image: a meta-analysis. J. Health Psychol. 14, 780–793. 10.1177/1359105309338977 19687115

[B11] Caspersen C. J. Powell K. E. Christenson G. M. (1985). Physical activity, exercise, and physical fitness: definitions and distinctions for health-related research. Public Health Rep. 100, 126–131. 3920711 PMC1424733

[B12] Castro O. Bennie J. Vergeer I. Bosselut G. Biddle S. J. (2020). How sedentary are university students? A systematic review and meta-analysis. Prev. Sci. 21, 332–343. 10.1007/s11121-020-01093-8 31975312

[B13] Centers for Disease Control (2008). Physical activity guidelines for Americans. Available online at: https://www.cdc.gov (accessed September 28, 2020).

[B14] Chan J. S. Y. Liu G. Liang D. Deng K. Wu J. Yan J. H. (2019). Special issue—therapeutic benefits of physical activity for mood: a systematic review on the effects of exercise intensity, duration, and modality. J. Psychol. 153, 102–125. 10.1080/00223980.2018.1470487 30321106

[B15] Chastin S. F. Egerton T. Leask C. Stamatakis E. (2015). Meta-analysis of the relationship between breaks in sedentary behavior and cardiometabolic health. Obesity 23, 1800–1810. 10.1002/oby.21180 26308477

[B16] Chekroud S. R. Gueorguieva R. Zheutlin A. B. Paulus M. Krumholz H. M. Krystal J. H.. (2018). Association between physical exercise and mental health in 1·2 million individuals in the USA between 2011 and 2015: a cross-sectional study. Lancet Psychiatry 5, 739–746. 10.1016/S2215-0366(18)30227-X 30099000

[B17] Chrisman J. A. Chambers Christopher J. Lichtenstein S. J. (2009). Qigong as a mindfulness practice for counseling students: a qualitative study. J. Humanist Psychol. 49, 236–257. 10.1177/00221678083277506668417

[B18] Cohen J. (1988). Statistical Power Analysis for the Behavioral Sciences, 2nd ed. Hillsdale, NJ: Erlbaum.

[B19] Cohen S. Kamarck T. Mermelstein R. (1983). A global measure of perceived stress. J. Health Soc. Behav. 24, 385–396. 10.2307/2136404 6668417

[B20] Cool J. Zappetti D. (2019). The Physiology of Stress, in Medical Student Well-Being, eds Zappetti D. Avery J. (Cham: Springer), 1–15. 10.1007/978-3-030-16558-1_1

[B21] Corbin C. B. Pangrazi R. P. Franks B. D. (2000). Definitions: health, fitness, and physical activity. Pres. Counc. Phys. Fit. Sports Res. Dig. 3, 1–11. http://eric.ed.gov/?id=ED470696 PMC3022443 21253445

[B22] de Bruin E. I. Formsma A. R. Frijstein G. Bögels S. M. (2017). Mindful2Work: effects of combined physical exercise, yoga, and mindfulness meditations for stress relieve in employees: a proof of concept study. Mindfulness 8, 204–217. 10.1007/s12671-016-0593-x 28163797 PMC5241323

[B23] Deckro G. R. Ballinger K. M. Hoyt M. Wilcher M. Dusek J. Myers P.. (2010). The evaluation of a mind/body intervention to reduce psychological distress and perceived stress in college students. J. Am. Coll. Health 50, 281–287. 10.1080/07448480209603446 12701653

[B24] Dempsey P. C. Owen N. Biddle S. J. Dunstan D. W. (2014). Managing sedentary behavior to reduce the risk of diabetes and cardiovascular disease. Curr. Diab. Rep. 14:522. 10.1007/s11892-014-0522-0 25052856

[B25] Dolbier C. L. Rush T. E. (2012). Efficacy of abbreviated progressive muscle relaxation in a high-stress college sample. Int. J. Stress Manag. 19, 48–68. 10.1037/a002732627168479

[B26] Drummy C. Murtagh E. M. McKee D. P. Breslin G. Davison G. W. Murphy M. H. (2016). The effect of a classroom activity break on physical activity levels and adiposity in primary school children. J. Paediatr. Child Health 52, 745–749. 10.1111/jpc.13182 27168479

[B27] Eisenberg D. Nicklett E. J. Roeder K. Kirz N. E. (2011). Eating disorder symptoms among college students: prevalence, persistence, correlates, and treatment-seeking. J. Am. Coll. Health 59, 700–707. 10.1080/07448481.2010.546461 21950250 PMC3721327

[B28] Engeroff T. Ingmann T. Banzer W. (2018). Physical activity throughout the adult life span and domain-specific cognitive function in old age: a systematic review of cross-sectional and longitudinal data. Sports Med. 48, 1405–1436. 10.1007/s40279-018-0920-6 29667159

[B29] Faul F. Erdfelder E. Buchner A. Lang A.-G. (2009). Statistical power analyses using G^*^Power 3.1: tests for correlation and regression analyses. Beh. Res. Methods 41, 1149–1160. 10.3758/BRM.41.4.1149 19897823

[B30] Feter N. dos Santos T. S. Caputo E. L. da Silva M. C. (2019). What is the role of smartphones on physical activity promotion? A systematic review and meta-analysis. Int. J. Health 64, 679–690. 10.1007/s00038-019-01210-7 30758514

[B31] Field A. Miles J. Field Z. (2012). Discovering statistics using R. 1st ed. London: SAGE Publications.

[B32] Fredrickson B. L. (1998). What good are positive emotions? Rev. Gen. Psychol. 2, 300–319. 10.1037/1089-2680.2.3.300 21850154 PMC3156001

[B33] Frisina P. G. Borod J. C. Lepore S. J. (2004). A meta-analysis of the effects of written emotional disclosure on the health outcomes of clinical populations. J. Nerv. Ment. Dis. 192, 629–634. 10.1097/01.nmd.0000138317.30764.63 15348980

[B34] Galante J. Dufour G. Vainre M. Wagner A. P. Stochl J. Benton A.. (2018). A mindfulness-based intervention to increase resilience to stress in university students (the Mindful Student Study): a pragmatic randomised controlled trial. Lancet Public Health 3, e72–e81. 10.1016/S2468-2667(17)30231-1 29422189 PMC5813792

[B35] Garner D. M. (1991). Eating disorder Inventory-2: professional manual. Odessa, FL: Psychological Assessment Ressources.

[B36] Gerber M. Brand S. Herrmann C. Colledge F. Holsboer-trachsler E. Pühse U. (2014). Increased objectively assessed vigorous-intensity exercise is associated with reduced stress, increased mental health and good objective and subjective sleep in young adults. Physiol. Behav. 135, 17–24. 10.1016/j.physbeh.2014.05.047 24905432

[B37] Gibson A.-M. Muggeridge D. J. Hughes A. R. Kelly L. Kirk A. (2017). An examination of objectively-measured sedentary behavior and mental well-being in adults across week days and weekends. PLoS ONE 3, 1–9. 10.1371/journal.pone.0185143 28934319 PMC5608355

[B38] Gill D. L. Hammond C. C. Reifsteck E. J. Jehu C. M. Williams R. A. Adams M. M.. (2013). Physical activity and quality of life. J. Prev. Med. Public Health 46, 28–34. 10.3961/jpmph.2013.46.S.S28 23412703 PMC3567315

[B39] Grobe T. G. Steinmann S. Szecsenyi J. (2018). BARMER Arztreport 2018 Schriftenreihe zur Gesundheitsanalyse, 7th ed. Berlin: BARMER.

[B40] Gronwald T. Velasques B. Ribeiro P. Machado S. Murillo-Rodríguez E. Ludyga S.. (2018). Increasing exercise's effect on mental health: exercise intensity does matter [Letter to the editor]. Proc. Natl. Acad. Sci U.S.A 115, E11890–E11891. 10.1073/pnas.1818161115 30568027 PMC6304946

[B41] Grützmacher J. Gusy B. Lesener T. Sudheimer S. Willige J. (2017). Gesundheit Studierender in Deutschland 2017. Ein Kooperationsprojekt zwischen dem Deutschen Zentrum für Hochschul- und Wissenschaftsforschung, der Freien Universität Berlin und der Techniker Krankenkasse, Hannover.

[B42] Hall G. Laddu D. R. Phillips S. A. Lavie C. J. Arena R. (2020). A tale of two pandemics: how will COVID-19 and global trends in physical inactivity and sedentary behavior affect one another? Prog. Cardiovasc. Dis. 64, 108–110. 10.1016/j.pcad.2020.04.005 32277997 PMC7194897

[B43] Hallgren M. Nguyen T. T. Owen N. Vancampfort D. Smith L. Dunstan D. W.. (2020). Associations of interruptions to leisure-time sedentary behavior with symptoms of depression and anxiety. Transl. Psychiatry 10, 1–8. 10.1038/s41398-020-0810-1 32366824 PMC7198536

[B44] Hammami A. Harrabi B. Mohr M. Krustrup P. (2020). Physical activity and coronavirus disease 2019 (COVID-19): specific recommendations for home-based physical training. Manag. Sport Leis. 1–6. 10.1080/23750472.2020.1757494

[B45] Hammen C. (2005). Stress and depression. Annu. Rev. Clin. Psychol. 1, 293–319. 10.1146/annurev.clinpsy.1.102803.143938 17716090

[B46] Hansen B. H. Home I. Anderssen S. A. Kolle E. (2013). Patterns of objectively measured physical activity in normal weight, overweight, and obese individuals (20–85 years): a cross-sectional study. PLoS ONE 8:e53044. 10.1371/journal.pone.0053044 23308135 PMC3538675

[B47] Hautzinger M. Keller F. Kühner C. (2006). Beck Depressions-Inventar (BDI-II): Revision. Frankfurt/Main: Harcourt Test Services.

[B48] Herbert C. Bendig E. Rojas R. (2019). My sadness–our happiness: writing about positive, negative, and neutral autobiographical life events reveals linguistic markers of self-positivity and individual well-being. Front. Psychol. 9:2522. 10.3389/fpsyg.2018.02522 30670993 PMC6331680

[B49] Herbert C. Gilg V. Sander M. Kobel S. Jerg A. Steinacker J. M. (2020a). Preventing mental health, well-being and physical activity during the corona pandemic: recommendations from psychology and sports medicine. German J. Sports Med. 71, 249–257. 10.5960/dzsm.2020.45832528333

[B50] Herbert C. Meixner F. Wiebking C. Gilg V. (2020b). Regular physical activity, short-term exercise, mental health, and well-being among university students: the results of an online and a laboratory study. Front. Psychol. 11:509. 10.3389/fpsyg.2020.00509 32528333 PMC7264390

[B51] Huang J. Nigatu Y. T. Smail-Crevier R. Zhang X. Wang J. (2018). Interventions for common mental health problems among university and college students: a systematic review and meta-analysis of randomized controlled trials. J. Psychiatr. Res. 107, 1–10. 10.1016/j.jpsychires.2018.09.018 30300732

[B52] Hubbard K. K. Blyler D. (2016). Improving academic performance and working memory in health science graduate students using progressive muscle relaxation training. Am. J. Occup. Ther. 70:7006230010. 10.5014/ajot.2016.020644 27767946

[B53] Irwin J. D. (2004). Prevalence of university students' sufficient physical activity: a systematic review. Percept. Mot. Skill. 98, 927–943. 10.2466/pms.98.3.927-943 15209309

[B54] Islam M. A. Barna S. D. Raihan H. Khan M. N. A. Hossain M. T. (2020). Depression and anxiety among university students during the COVID-19 pandemic in Bangladesh: a web-based cross-sectional survey. PloS ONE 15:e0238162. 10.1371/journal.pone.0238162 32845928 PMC7449469

[B55] Jacobson E. (1938). Progressive muscle relaxation. J. Abnorm. Psychol. 75:18.20726214

[B56] Jadoon N. A. Yaqoob R. Raza A. Shehzad M. A. Choudhry Z. S. (2010). Anxiety and depression among medical students: a cross-sectional study. J. Pak. Med. Assoc. 60, 699–702. 20726214

[B57] Kohl H. W. Craig C. L. Lambert E. V. Inoue S. Alkandari J. R. Leetongin G.. (2012). The pandemic of physical inactivity: global action for public health. Lancet 380, 294–305. 10.1016/S0140-6736(12)60898-8 22818941

[B58] Kramer A. (2020). An overview of the beneficial effects of exercise on health and performance. in Physical Exercise for Human Health, ed J. Xiao (Singapore: Springer), 3–22. 10.1007/978-981-15-1792-1_1 32342447

[B59] Krohne H. W. Egloff B. Kohlmann C.-W. Tausch A. (1996). Untersuchungen mit einer deutschen Version der Positive and Negative Affect Schedule (PANAS). Diagnostica 42, 139–156. 10.1037/t49650-000

[B60] Krpan K. M. Kross E. Berman M. G. Deldin P. J. Askren M. K. Jonides J. (2013). An everyday activity as a treatment for depression: the benefits of expressive writing for people diagnosed with major depressive disorder. J. Affect. Disord. 150, 1148–1151. 10.1016/j.jad.2013.05.065 23790815 PMC3759583

[B61] Lima M. M. S. Vieira A. P. (2007). Ballroom dance as therapy for the elderly in Brazil. Am. J. Dance Ther. 29, 129–142. 10.1007/s10465-007-9040-932270698

[B62] Lippi G. Henry B. M. Sanchis-Gomar F. (2020). Physical inactivity and cardiovascular disease at the time of coronavirus disease 2019 (Covid-19). Eur. J. Prev. Cardiol. 27, 906–908. 10.1177/2047487320916823 32270698 PMC7717305

[B63] López-Bueno R. Calatayud J. Andersen L. L. Balsalobre-Fernández C. Casaña J. Casajús J. A.. (2020). Immediate impact of the Covid-19 confinement on physical activity levels in Spanish adults. Sustainability 12:5708. 10.3390/su1214570828590767

[B64] López-Rodríguez M. M. Baldrich-Rodríguez I. Ruiz-Muelle A. Cortés-Rodríguez A. E. Lopezosa-Estepa T. Roman P. (2017). Effects of biodanza on stress, depression, and sleep quality in university students. J. Altern. Complement. Med. 23, 558–565. 10.1089/acm.2016.0365 28590767

[B65] Mair P. Wilcox R. R. (2020). Robust statistical methods in R using the WRS2 package. Behav. Res. Methods 52, 464–488. 10.3758/s13428-019-01246-w 31152384

[B66] Martinsen E. W. Hoffart A. Solberg Ø. (1989a). Comparing aerobic with nonaerobic forms of exercise in the treatment of clinical depression: a randomized trial. Compr. Psychiatry 30, 324–331. 10.1016/0010-440X(89)90057-6 2667882

[B67] Martinsen E. W. Hoffart A. Solberg Ø. (1989b). Aerobic and non-aerobic forms of exercise in the treatment of anxiety disorders. Stress Med. 5, 115–120. 10.1002/smi.246005020932613133

[B68] Maugeri G. Castrogiovanni P. Battaglia G. Pippi R. D'Agata V. Palma A.. (2020). The impact of physical activity on psychological health during Covid-19 pandemic in Italy. Heliyon 6:e04315. 10.1016/j.heliyon.2020.e04315 32613133 PMC7311901

[B69] Mikkelsen K. Stojanovska L. Polenakovic M. Bosevski M. Apostolopoulos V. (2017). Exercise and mental health. Maturitas 106, 48–56. 10.1016/j.maturitas.2017.09.003 29150166

[B70] Murtagh E. Mulvihill M. Markey O. (2013). Bizzy Break! The effect of a classroom-based activity break on in-school physical activity levels of primary school children. Pediatr. Exerc. Sci. 25, 300–307. 10.1123/pes.25.2.300 23504941

[B71] Musch J. Brockhaus R. Bröder A. (2002). Ein Inventar zur Erfassung von zwei Faktoren sozialer Erwünschtheit. Diagnostica 48, 121–129. 10.1026//0012-1924.48.3.12124708352

[B72] Park D. Ramirez G. Beilock S. L. (2014). The role of expressive writing in math anxiety. J. Exp. Psychol. Appl. 20, 103–111. 10.1037/xap0000013 24708352

[B73] Paul T. Thiel A. (2005). EDI-2: Eating Disorder Inventory-2. Hogrefe: Deutsche Version.

[B74] Paulhus D. L. (1988). Assessing self deception and impression management in self-reports: the Balanced Inventory of Desirable Responding. Vancouver, BC: University of British Columbia. Canada.

[B75] Pennebaker J. W. (2018). Expressive writing in psychological science. Perspect. Psychol. Sci. 13, 226–229. 10.1177/1745691617707315 28992443

[B76] Pennebaker J. W. Beall S. K. (1986). Confronting a traumatic event: toward an understanding of inhibition and disease. J. Abnorm. Psychol. 95, 274–281. 10.1037/0021-843X.95.3.274 3745650

[B77] Pennebaker J. W. Chung C. (2007). Expressive writing, emotional upheavals, and health, in Foundations of Health Psychology, eds Friedman H. S. Silver R. C. (New York, NY: Oxford University Press), 263–284.

[B78] Pennebaker J. W. Francis M. E. Booth R. J. (2001). Linguistic Inquiry and Word Count: LIWC 2001. Mahway, NJ: Lawrence Erlbaum Associates.

[B79] Pinheiro J. Bates D. DebRoy S. Sarkar D. R Core Team (2019). nlme: Linear and Nonlinear mixed effects models. Retrieved from R package version 3.1-143. Available online at: https://cran.r-project.org/package=nlme (accessed September 28, 2020).

[B80] Pitt A. Oprescu F. Tapia G. Gray M. (2018). An exploratory study of students' weekly stress levels and sources of stress during the semester. Act. Learn. High. Educ. 19, 61–75. 10.1177/1469787417731194

[B81] Powell K. E. King A. C. Buchner D. M. Campbell W. W. DiPietro L. Erickson K. I.. (2019). The scientific foundation for the physical activity guidelines for Americans. 2nd ed. J. Phys. Act. Health 16, 1–11. 10.1123/jpah.2018-0618 30558473

[B82] Questback GmbH (2016). EFS Survey, Version Herbst 2016. Köln: Questback GmbH.

[B83] R Studio Team (2019). RStudio: integrated development for R. Available online at http://www.rstudio.com/ (Accessed September 28, 2020).

[B84] Radwan H. Hasan H. A. Ismat H. Hakim H. Khalid H. Al-Fityani L.. (2019). Body mass index perception, body image dissatisfaction and their relations with weight-related behaviors among university students. Int. J. Environ. Res. Public Health 16:1541. 10.3390/ijerph16091541 PMC6539402 31052368

[B85] Regehr C. Glancy D. Pitts A. (2013). Interventions to reduce stress in university students: a review and meta-analysis. J. Affect. Disord. 148, 1–11. 10.1016/j.jad.2012.11.026 23246209

[B86] Rueggeberg R. Wrosch C. Miller G. E. (2012). The different roles of perceived stress in the association between older adults' physical activity and physical health. Health Psychol. 31, 164–171. 10.1037/a0025242 21875206 PMC3279628

[B87] Ryan R. M. Frederick C. M. Lepes D. Rubio N. Sheldon K. M. (1997). Intrinsic motivation and exercise adherence. Int. J. Sport Psychol. 28, 335–354.15025890

[B88] Sancier K. M. Holman D. (2004). Multifaceted health benefits of medical qigong. J. Altern. Complement. Med. 10, 163–166. 10.1089/107555304322849084 15025890

[B89] Satow L. (2012). *Stress- und Coping-Inventar (SCI)* [PSYNDEX Tests-Nr. 9006508], ed. Leibniz-Zentrum für Psychologische Information und Dokumentation (ZPID), Elektronisches Testarchiv. Trier: ZPID.

[B90] Shepard R. J. (1988). PAR-Q: Canadian Home Fitness Test and exercise screening alternatives. Sports Med. 5, 185–195. 10.2165/00007256-198805030-00005 3368685

[B91] Silberstein L. R. Striegel-Moore R. H. Timko C. Rodin J. (1988). Behavioral and psychological implications of body dissatisfaction: do men and women differ? Sex Roles 19, 219–232. 10.1007/BF0029015618410204

[B92] Sloan D. M. Marx B. P. Epstein E. M. Dobbs J. L. (2008). Expressive writing buffers against maladaptive rumination. Emotion 8, 302–306. 10.1037/1528-3542.8.2.302 18410204

[B93] Strath S. J. Kaminsky L. A. Ainsworth B. E. Ekelund U. Freedson P. S. Gary R. A.. (2013). On behalf of the American Heart Association Physical Activity Committee of the Council on Lifestyle and Cardiometabolic Health, Exercise, Cardiac Rehabilitation and Prevention Committee of the Council on Clinical Cardiology, and Council on Cardiovascular and Stroke Nursing. Guide to the assessment of physical activity: clinical and research applications: a scientific statement from the American Heart Association. Circulation 128, 2259–2279. 10.1161/01.cir.0000435708.67487.da 24126387

[B94] Techniker Krankenkasse (2016). Beweg Dich, Deutschland! TK-Bewegungsstudie 2016. Hamburg: Techniker Krankenkasse.

[B95] The WHOQOL Group (1998). Development of the World Health Organization WHOQOL-BREF Quality of Life assessment. Psychol. Med. 28, 551–558. 10.1017/S0033291798006667 9626712

[B96] Thiel A. Jacobi C. Horstmann S. Paul T. Nutzinger D. O. Schüssler G. (1997). A German version of the Eating Disorder Inventory EDI-2. Psychother. Psychosom. Med. Psychol. 47, 365–376. 9411465

[B97] Tremblay M. S. Colley R. C. Saunders T. J. Healy G. N. Owen N. (2010). Physiological and health implications of a sedentary lifestyle. Appl. Physiol. Nutr. Metab. 35, 725–740. 10.1139/H10-079 21164543

[B98] Wankel L. M. (1993). The importance of enjoyment to adherence and psychological benefits from physical activity. Int. J. Sport Psychol. 24, 151–169.3397865

[B99] Watson D. Clark L. A. Tellegen A. (1988). Development and validation of brief measures of positive and negative affect: the PANAS scales. J. Pers. Soc. Psychol. 54, 1063–1070. 10.1037/0022-3514.54.6.1063 3397865

[B100] Widström A. M. Lilja G. Aaltomaa-Michalias P. Dahllöf A. Lintula M. Nissen E. (2011). Newborn behaviour to locate the breast when skin-to-skin: a possible method for enabling early self-regulation. Acta Paediatr. Int. J. Paediatr. 100, 79–85. 10.1111/j.1651-2227.2010.01983.x 20712833

[B101] Wilcox R. R. (2017). Introduction to robust estimation and hypothesis testing, 4th ed. San Diego, CA: Academic Press. 10.1016/B978-0-12-804733-0.00010-X

[B102] Woods J. A. Hutchinson N. T. Powers S. K. Roberts W. O. Gomez-Cabrera M. C. Radak Z.. (2020). The Covid-19 pandemic and physical activity. Sport Med. Health Sci. 2, 55–64. 10.1016/j.smhs.2020.05.006 PMC7261095 34189484

[B103] World Health Organization (2010). Global Recommendations on Physical Activity for Health. Geneva, WHO. 26180873

[B104] Wunsch K. Wurst R. von Dawans B. Strahler J. Kasten N. Fuchs R. (2019). Habitual and acute exercise effects on salivary biomarkers in response to psychosocial stress. Psychoneuroendocrinology 106, 216–225. 10.1016/j.psyneuen.2019.03.015 31003138

