# Supplementary material for: A Short, Multimodal Activity Break Incorporated Into the Learning Context During the Covid-19 Pandemic: Effects of Physical Activity and Positive Expressive Writing on University Students' Mental Health—Results and Recommendations From a Pilot Study
Source: Front Psychol. 2021 Aug 12;12:645492. doi: 10.3389/fpsyg.2021.645492 (PMC8397384; doi:10.3389/fpsyg.2021.645492)
Supplement: Supplementary file 1 [file Data_Sheet_1.pdf]

A Short, Multimodal Activity Break Incorporated Into the Learning Context During the Covid-19 Pandemic: Effects of Physical Activity and Positive Expressive Writing on University Students' Mental Health—Results and Recommendations From a Pilot Study

Verena Marschin and Cornelia Herbert

Supplementary Material

1 d2-R (Brickenkamp et al., 2010)

Two data rows were excluded completely from the data of the first test of the *d2-R Test for Attention* (Brickenkamp et al., 2010) for the baseline measurement (T0). Considering this exclusion, $N = 79$ students (physical activity group [PAG]: $n = 46$; cognitive intervention group [CG]: $n = 33$) enrolled in the study and filled out the d2 (Brickenkamp et al., 2010). $n = 1$ participant was further excluded from d2 data (Brickenkamp et al., 2010) because of late enrollment, hence no data for the baseline measurement was provided. The dropout rate in the d2 (Brickenkamp et al., 2010) for T3 was 75.95 % (EG: 78.26 %; CG: 72.73 %), $\chi^2(1) = 0.32$, $p = .570$, with odds of dropping out having been 1.35 (0.42, 4.32). A sample of $N = 18$ (PAG group: $n = 10$; CG: $n = 8$) students provided complete data for T0 and T3.

For the d2-R (Brickenkamp et al., 2010), mean age was significantly higher for the cognitive intervention group than for the physical activity group (PAG: $M_{age} = 22.50$, $SD_{age} = 1.43$; CG: $M_{age} = 26.25$, $SD_{age} = 6.43$), 95% CI [-11.92, -1.03].

Concentration values of the d2-R (Brickenkamp et al., 2010) were slightly higher for the PAG ($M_{T0} = 108.55$, $SD_{T0} = 9.40$; $M_{T3} = 116.10$, $SD_{T3} = 10.42$) compared to the CG ($M_{T0} = 105.56$, $SD_{T0} = 6.64$; $M_{T3} = 111.19$, $SD_{T3} = 9.47$). There was no significant group effect, $p > .05$. Values increased from T0 to T3. This effect of *time* was significant, $B = 9.75$, $SE_B = 2.35$, $t(16) = 4.15$, $p < .001$, $r = .72$. There was no significant effect for the interaction, $B = 0.65$, $SE_B = 3.16$, $t(16) = 0.21$, $p = .839$.

2 Reasons for Exercise Inventory (Silberstein et al., 1988)

Participants reported to exercise mainly for fitness reasons ($M = 20.80$, $SD = 5.34$) as measured via the *Reasons for Exercise Inventory* (REI; Silberstein et al., 1988), followed by health reasons ($M = 20.05$, $SD = 6.28$) and in order to improve mood ($M = 17.25$, $SD = 6.10$). Reasons stated as less important were weight correction ($M = 12.55$, $SD = 4.42$), enhancing attractiveness ($M = 11.50$, $SD = 4.24$), toning ($M = 11.25$, $SD = 4.01$) and enjoyment ($M = 10.50$, $SD = 4.43$).
